# Familiarity with an Object’s Size Influences the Perceived Size of Its Image

**DOI:** 10.3390/vision6010014

**Published:** 2022-02-24

**Authors:** Jeroen B. J. Smeets, Pauline E. Weijs, Eli Brenner

**Affiliations:** Department of Human Movement Sciences, Vrije Universiteit Amsterdam, NL-1081 BT Amsterdam, The Netherlands; pauline.weijs@gmail.com (P.E.W.); eli.brenner@vu.nl (E.B.)

**Keywords:** visual size, size perception, cue combination, expectations, Bayesian prior, precision, bias

## Abstract

It is known that judgments about objects’ distances are influenced by familiar size: a soccer ball looks farther away than a tennis ball if their images are equally large on the retina. We here investigate whether familiar size also influences judgments about the size of images of objects that are presented side-by-side on a computer screen. Sixty-three participants indicated which of two images appeared larger on the screen in a 2-alternative forced-choice discrimination task. The objects were either two different types of balls, two different types of coins, or a ball and a grey disk. We found that the type of ball biased the comparison between their image sizes: the size of the image of the soccer ball was over-estimated by about 5% (assimilation). The bias in the comparison between the two balls was equal to the sum of the biases in the comparisons with the grey disk. The bias for the coins was smaller and in the opposite direction (contrast). The average precision of the size comparison was 3.5%, irrespective of the type of object. We conclude that knowing a depicted object’s real size can influence the perceived size of its image, but the perceived size is not always attracted towards the familiar size.

## 1. Introduction

When interacting with the world around us, we use retinal size information to judge the distances and sizes of three-dimensional objects. The geometrical relationship between retinal image size and the object’s size and distance means that knowing its size or distance can help determine the other. It is known that judged distance is influenced by familiar size: a soccer ball looks farther away than a tennis ball when their images are equally large on the retina [1]. This illusion also influences how we interact with such an object: if a matchbox looks like that of a brand that sells large matchboxes, one grasps it as if it were a larger box that is positioned farther away [2]. In this situation, the effect of familiarity is an assimilation: objects are perceived in a way that corresponds to our expectations. Despite the textbook status of the effect of familiar size on perception of spatial relations, various authors have reported a lack of effect. This might be related to the way in which depth is interpreted in pictures of scenes [3] and thus to the instructions [4] and specifics of the task. More specifically, the effect of familiar size might be absent in direct comparisons [5] The question we address in the present paper is how prior knowledge about objects’ sizes influences judgments of the relative sizes of the objects’ isolated images when presented side-by-side on a screen under normal viewing conditions.

In experiments, one generally assumes that participants answer the question that the experimenter poses. However, questions can be understood or answered in various ways, leading to different answer [6,7]. When a moving observer judges the speed of an object, is it the speed relative to the observer or relative to the environment [8]? When judging the color of a sheet of paper, is it the color of the light reaching the eye or the reflectance of the paper that matters? [9]. Matching the light reaching the eye and matching surface reflectance are fundamentally different judgments, but subjects cannot always choose which to match. The issue of which question is answered might play a role in experiments on size perception: are participants reporting about the size of the object, the size of its retinal image (angular size), or a mixture of both?

When judging the size of the object it makes sense to consider everything that one knows about such an object’s usual size. Considering familiar size in this manner when judging an object’s size results in assimilation: the object is judged to be closer in size to the familiar size. The familiar size can be modeled as a Bayesian prior [10]. The result of considering the familiar size is not only that the average percept is in between the sensed and expected value, but also that the precision of the percept is improved. Thus, according to the Bayesian explanation for assimilation, one would predict that the comparison of two familiar objects would be more precise than comparing a familiar with a neutral object.

Before proceeding with the present experiment, we would like to note that the influence of expectations based on familiarity is not always assimilation. Large objects are generally heavier than small ones. Nevertheless, when we lift (or hit [11]) a tennis ball and a soccer ball of the same mass, the soccer ball does not feel heavier, but lighter (the size-weight illusion [12,13]). Contrast-effects such as the size-weight illusion can be interpreted as an anti-Bayesian combination of sensory information [14]. However, that interpretation assumes that participants report about the property that they are asked to judge. In the case of the size-weight illusion, participants might be inclined to report the mass per unit-volume (density) rather than the total mass, as this quantity corresponds better with the intuitive idea of weight [15,16]. If this is the case, the size-weight illusion would be Bayesian after all [15,17]. Although we have provided some experimental evidence against this interpretation of the size-weight illusion [18], the fact remains that identifying the attributes that a participant uses to answer the experimenter’s question is an important issue in perception research [6].

In the present experiment, we remove any influence of this ambiguity by asking participants to report which of two images on a computer screen is larger. By presenting images simultaneously on an empty screen in an illuminated room, rather than embedded within a scene or in isolation in the dark, it should be evident that the images are at the same distance so that reporting about their relative angular sizes or image sizes is equivalent to reporting about the relative sizes of the objects that are depicted on the screen. We use two sets of images: one set of images of balls and one of coins. A ball is a 3D object that we are used to view in various situations (above and below eye-height, on the ground or moving in the air), generally without other balls in the vicinity. A coin is a 2D object that generally lies below eye height (e.g., in a purse or on a counter) in the direct vicinity of other coins. For judging the size of a ball, we generally have fewer cues about their size than for a coin. We therefore expect a larger effect of familiar size for the balls than for the coins.

In the experiment, we address a second issue in judgements of size. It has been shown that the strength of the Ebbinghaus illusion as obtained by comparing two objects in opposite inducing environments differs from the strength as obtained by comparing each object in an illusory environment with a neutral object (super-additivity [19]). Besides investigating how familiar size influences judgments of the size of images that are presented on a computer screen (is it assimilation or contrast?) we also examined whether super-additivity plays a role in this judgement.

## 2. Methods

This experiment was programmed as an online web-application. It was built using the jsPsych javascript library [20]. Seventy participants had to indicate which of two objects appeared larger on the screen in a 2-alternative forced-choice discrimination task. The objects could be balls (tennis or soccer) or coins (10 eurocent or 2 euro). Participants also compared the images of the balls with a grey disk.

### 2.1. Participants

The experiment was performed as part of a research project for a master’s degree at the Vrije Universiteit Amsterdam and was therefore constrained by limitations in time. As we had to perform the experiment during the corona pandemic, we had to use on-line experimentation. The consequence is that we had no control of the physical size of the monitor and could also not control viewing distance. As we had no idea how these additional sources of variability would affect the reliability of the data, we did not perform a power analysis. Performing the experiment was not burdensome, so we tried to recruit as many participants as possible. Ultimately, we recruited 70 participants aged between 17 and 73 years old (median age 25, 37 female). Half of the participants were students who received study credits for their participation, the other half were volunteers recruited from the second author’s network. All reported to have normal or corrected to normal vision and gave their informed consent before they performed the experiment. Each participant completed the experiment at his/her own personal computer, and then sent the results back to the authors. This research is part of a research program that is approved by the Vaste Commissie Wetenschap en Ethiek van de Faculteit der Gedrags- en Bewegingswetenschappen.

### 2.2. Stimuli and Procedure

**Figure 1:**
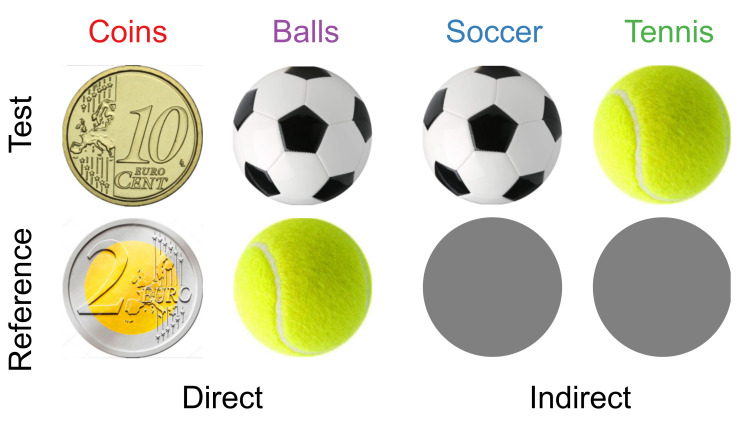
Overview of the stimuli that we used in the four conditions of the experiment. In the two *Direct* conditions, two images were presented (either Balls or Coins); in the two *Indirect* conditions, a ball was presented together with a grey reference disk. Note that in the analysis of the Coins condition, test and reference are switched when interpreting the size difference (see for instance insets of Figure 2) to make it easier to compare the curves.

We used a 2-alternative forced choice task to test whether familiar size plays a role in size estimation. On each trial, the participant’s task was to determine which of two images was the largest. We used a total of five different kinds of circular images (Figure 1). Four of them were images of familiar objects of a well-defined size. Two images of balls (a tennis ball and a soccer ball), objects that are three-dimensional in the real world. The other two were images of coins (€0.10 and €2), objects that are flat in the real world. The fifth image was a uniform grey disk, not corresponding to any real-life object. All images were presented on a uniform white background. To facilitate judging the actual image size on the screen, we allowed participants to use all cues about the images’ distances [21]: participants viewed the images on a clearly visible screen, with two eyes and no restrictions on their eye or head movements. All dimensions of the experiment scale with the (unknown) screen size of each participant; the values we present here are approximations for a 14″ screen. The images were about 5 cm in diameter, presented simultaneously on a white background 15 cm apart. This distance, combined with the circular shape of the objects, made it very difficult to compare the upper and lower outlines across the two images. The objects were about 8 cm from the screen edges. Based on an estimate of the expected effect we obtained in a pilot experiment, we presented size differences ranging from −10% to +10%, with increments of 2.5% (9 sizes). As screen sizes will have varied between participants, we express the size of the test stimulus as a fraction of that of the reference throughout this paper. The physical euro coins have a precisely known diameter: 1.975 cm for the €0.10 coin and 2.575 cm for the €2 coin. There is some variation in real-world ball sizes. The typical diameter of a tennis ball is about 6.7 cm, whereas a size 5 soccer ball (used for players aged 13 and older) is about three times as large: 22 cm. So, the image of the soccer ball is always smaller than a real-world soccer ball, the image of the tennis ball is smaller than that of a real tennis ball unless the participant has a 19” screen or larger, and the images of the two types of coins are larger than the real-world objects (unless participants used a 7″ screen or smaller).

The comparisons are limited to four conditions. Two conditions were designed to determine the effect of familiar size on the judgement of image sizes of Balls and Coins. In these conditions, one image was the reference that always had the same size (€2 coin and tennis ball), whereas the other, test image could have one of various sizes. As a pilot experiment showed a larger effect of familiar size in the Balls condition, we used this condition to check whether a super-additive effect occurs when two balls are presented simultaneously [19]. To do so, we compared the images of the balls with the grey disk. In these conditions (Soccer and Tennis), the grey disk was the reference. If there is no super-additivity, the difference between the illusion effects obtained in the Soccer and Tennis conditions should cancel out the contribution of the grey disk, and thus equal the effect found in the Balls condition.

Combining the four conditions and nine sizes resulted in 36 object pairs. As the screen side might have an effect on the illusion strength [22], each object in these pairs could be displayed at the left or the right side of the screen. These two variants were each presented five times, resulting in a total of 360 trials.

To be sure that a possible difference between the Balls condition and the corresponding indirect conditions is not due to an order effect, we presented these three conditions in a single block. The Coins condition was presented in a separate block. The order of the blocks was counterbalanced across participants. Within each block, all trials appeared in random order. At the start of a block, the participants received this instruction on their screen: “If the object on the left side of the screen is larger than the object on the right side, press the ‘, ’ on the keyboard. If the object on the right side of the screen is larger, press the ‘.’ key”. In the formulation to the participant, we used ‘object’ for what we refer to as ‘image’ in the rest of the paper, but it was evident from the context that the participants were to compare the sizes of the images. We debriefed some of the volunteer participants, and all of them reported that they compared the size of the objects’ images on the screen. Participants could view the stimuli as long as they liked. After each response, the next stimulus appeared. Although not explicitly mentioned as a possibility, participants were able to take a break between trials by simply delaying their response. The median time to complete the experiment was 15 min.

### 2.3. Data Analysis

All analysis was performed in RStudio 1.4 (Rstudio, 2021, R Core Team, Boston, MA, USA). We used the quickpsy package [23] to fit a cumulative normal distribution to the proportion of trials in which the test stimulus was judged to be larger than the reference for each depicted size. We performed the analysis per participant and condition, assuming no lapses in the judgements were made [24]. We calculated 95% confidence intervals of the means (bias) and standard deviations (precision) using bootstrapping [25]. As we could not check whether participants had understood the task during the experiment, we checked each participant’s overall performance by checking whether their Weber fraction for size judgements was below 20%, i.e., whether their standard deviation was smaller than the range of sizes we presented. Reported Weber fractions for size judgements are clearly better than 10% [26,27,28,29,30]. We determined this Weber fraction for the pooled data of each participant, without differentiating between conditions. We also ensured that we could determine the bias reliably for each condition (a 95% confidence interval that is smaller than the range of stimuli).

In the design of the experiment, we chose arbitrarily which object served as the reference in the direct comparisons. To make the results easier to interpret, we inverted the sign of the judgements of the coins (i.e., regarded the €0.10 image as the reference). After this correction, a negative bias corresponds in all conditions to an assimilation effect of familiar size.

To test whether comparing two familiar objects differed from comparing a familiar to a neutral object, we compared the illusion effects found in the direct comparison (Balls condition) with that of the indirect conditions. For the latter we used the difference in biases between the Tennis and Soccer condition. As the data were not normally distributed, we used a Wilcoxon’s signed rank test to find out whether both illusion effects differed from each other and from zero. We used the same test to see whether the precision depended on the comparison method.

To explore whether performance in one condition was related to that in another condition, we determined the correlation across participants, both for the biases and the variability. To visualize the dependencies, we performed a weighted orthogonal regression for each combination of conditions. We chose an orthogonal regression because there was equal measurement error for both the ‘dependent’ as well as the ‘independent’ variable, whereas a simple linear regression model only takes a measurement error of the dependent variable into account. To ensure that participants’ influence on the regression is in line with the confidence in that participant’s data, we weighted each participant by 1 divided by the square root of the sum of squared lengths of the confidence intervals. We obtained the 95% confidence intervals of the model’s slope by bootstrapping.

As we managed to get a large group of participants, we include an exploratory analysis on whether the results depend on the age of the participants and whether it matters whether the participants performed the experiment in exchange for course-credit or volunteered to help the experimenter. We plotted the precision as a function of age for both groups.

## 3. Results

We removed four participants from further analysis because they performed extremely poorly: they had an overall Weber fraction larger than 20%. We removed three additional participants because we could not reliably determine their bias in one or more conditions. All these participants had performed the experiment in exchange for course-credit. The average precision (Weber fraction) of the size comparison for the remaining 63 participants was about 3.5%, irrespective of the objects.

**Figure 2:**
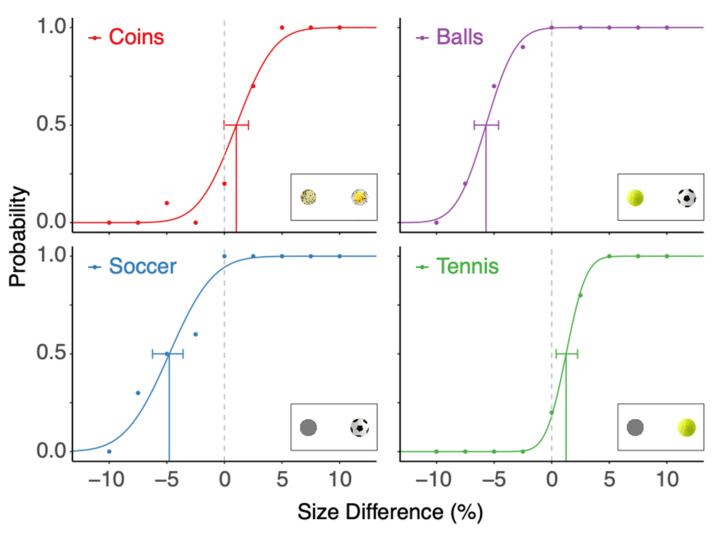
Results of an example participant. The probability of seeing the right image as larger is plotted as a function of the difference in size. Dots are the data; the curve is the cumulative Gaussian that we fitted. The biases are indicated by a solid vertical line, and the horizontal error bars are its corresponding 95% confidence intervals. The inset on the lower right of each panel shows an image corresponding to a 10% size difference in that condition.

The results of an example participant (Figure 2) show negative biases for the Soccer and Balls condition, which corresponds to a smaller image of a soccer ball looking as large as the other image. For the soccer ball, familiar size affects size perception in the direction of assimilation: the size of an image of a soccer ball that is smaller than its real-world size is overestimated. In contrast, this participant judged a tennis ball to be 1.1% smaller than an equally sized grey disk, although a real tennis ball is probably larger than the image on the screen (unless this participant had a 19″ screen or larger), so this was a contrast effect. Such a contrast effect also holds for the Coins condition: an equally sized image of a €0.10 coin was judged to be slightly larger than that of a €2 coin. The precision of this participant was best (lowest Weber-fraction, corresponding to the steepest slope) in the Tennis condition, and it was poorest in the Soccer condition.

**Figure 3:**
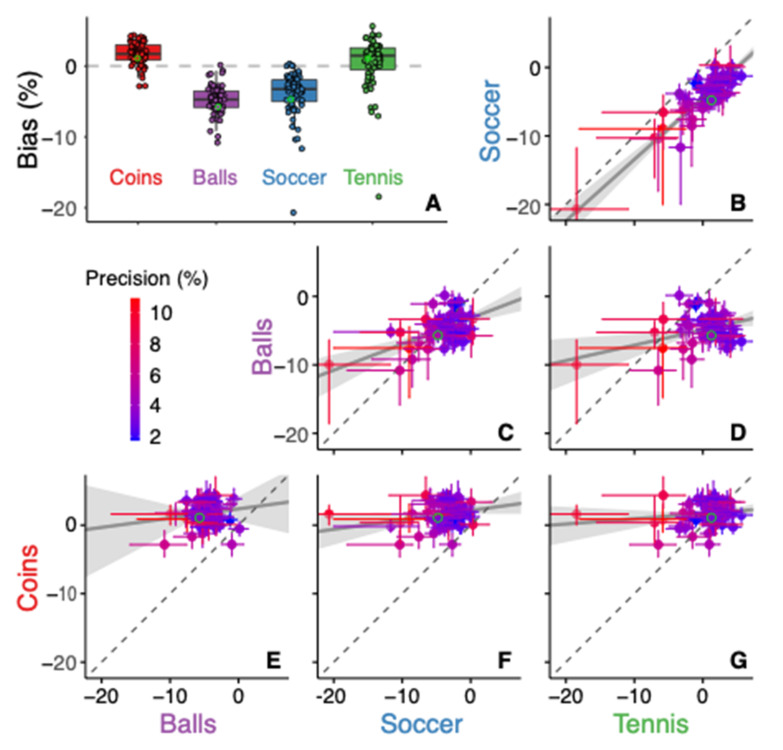
Participants’ individual biases in the four conditions. A negative bias corresponds to an assimilation effect of familiar size. The green outlines identify the data of the example participant of Figure 2. (**A**) Individual participants’ biases are plotted for the four conditions, superimposed on a boxplot. (**B**–**G**): the bias in one condition as a function of that in another condition. Dots with error bars represent individual biases with their 95% confidence intervals. Colour coding reflects the mean precision of the participant across all four conditions (see Figure 4). The dashed identity lines indicate the points with equal values for both conditions. The grey lines with shaded area indicate perpendicular regressions with their 95% confidence interval.

We found that familiar size systematically biased size perception across participants in a similar way as we saw for the example participant (Figure 3A): the assimilation in the Balls condition was present in all but one participant, and that in the Soccer condition in all but three participants, so the effect is significant in these conditions (*p* < 0.001). Overall, the size of the soccer ball was overestimated (assimilation) relative to the grey disk by about 5%, but the amount of overestimation varied considerably between participants. The bias when comparing the tennis ball with the grey disk was smaller, less consistent across participants, and on average in the opposite direction (contrast). The median bias of the tennis ball of 1.5% differed significantly from zero (*p* < 0.01), which indicates that the tennis ball was perceived as being smaller than the grey disc. When comparing the two balls, the bias corresponds to the difference between the Soccer and Tennis condition. The bias for the Coins condition was clearly different than for the Balls one: it was in the opposite direction (contrast) and smaller. The median bias of the Coins condition is 1.7%, which differs significantly from zero (*p* < 0.001). The positive bias indicates that the €0.10 coin was perceived as larger than the €2 coin, whereas its real-world size is smaller.

To explore a possible relation between the different conditions we plotted the individual data and a corresponding orthogonal fit in Figure 3B–G. The participants whose biases deviated the most from the mean were generally not very precise (red colors). There is a clear relation between the biases obtained from the Soccer and Tennis condition (Figure 3B), which were both comparisons to a grey disc. In comparison to the unity line, the orthogonal fit is shifted right/downwards, which corresponds to a difference in bias between these conditions that is independent of the magnitudes of the biases. When comparing the Tennis and Soccer condition with the Balls condition (Figure 3C,D), the correlation between the biases is much smaller. There is no correlation between the bias in the Coins condition and that in any of the other three conditions (Figure 3E–G). The strong correlation between the Soccer and Tennis conditions might be related to the presence of the grey disc. If participants have idiosyncratic priors for the size of the grey disc, this could be a common source of variance in these two conditions. This common source of variance not only explains the correlation, but also why the variability between participants in these conditions is larger than in the Balls condition.

**Figure 4:**
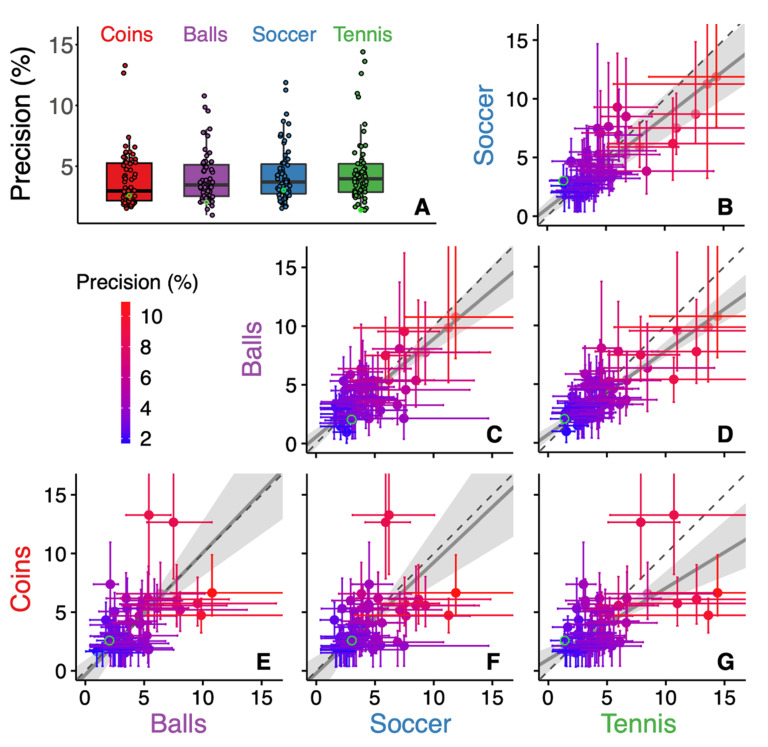
Participants’ individual precision in the four conditions. (**A**) Individual participants’ data are plotted for the four conditions, superimposed on a boxplot. (**B**–**G**): the precision in one condition as a function of that in another condition. Dots with error bars represent individual precision with their 95% confidence intervals. See Figure 3 for further details.

The precision of the judgments varied considerably across participants (ranging from 1.7% to 10.9%) but was consistent across conditions (the identity line is within the 95% confidence interval of most datapoints of Figure 4B–G). The colour coding in Figure 3B–G shows that large biases are mostly found for participants who are not very precise (red symbols). Those large biases are probably not only due to random variability, because in some cases the correlations between the biases are clearly consistent across conditions (Figure 3B). This finding might therefore reflect the fact that participants who were less good at visually judging size (possibly even simply because they had smaller screens or sat further from them) relied to a greater extent on their familiarity with the object’s normal size.

**Figure 5:**
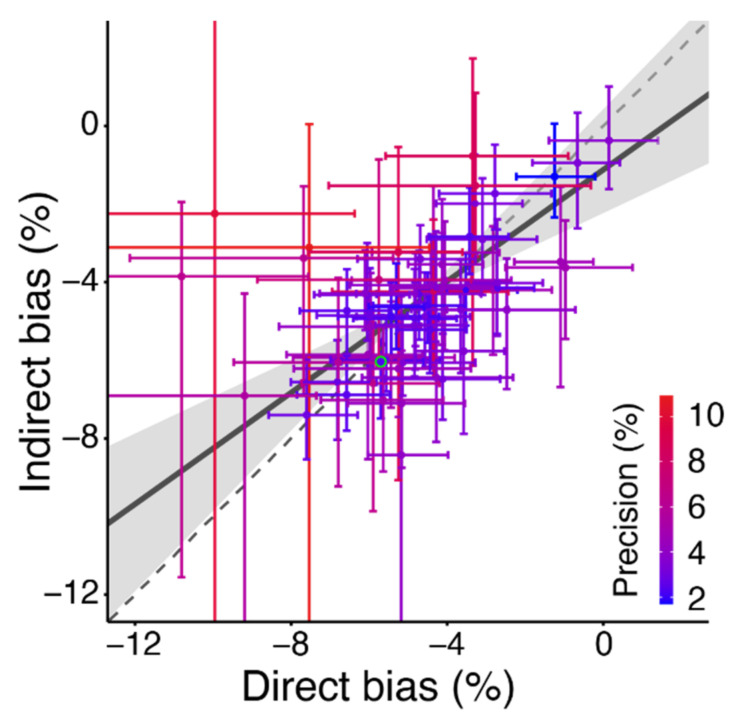
The bias in the comparison of the tennis ball and the soccer ball. We plotted the bias obtained from comparing the balls separately with neutral grey discs (Indirect) as a function of the bias obtained by comparing simultaneously presented images of the two objects (Direct). See Figure 3 for further details.

To find out whether a super-additive effect may have occurred in the balls condition, we compared the illusion effect of −4.3% obtained from the difference in bias between the Soccer and Tennis conditions (indirect bias) with the −4.7% direct bias of the Balls condition. The advantage of comparing the balls directly is small and not systematic (*p* = 0.57; Figure 5), with almost all participants’ biases being within their 95% confidence interval from the identity line. The slope of the fitted line is close to that of the identity line, so participants that have a larger bias than average in the direct comparisons do also have larger biases in the indirect ones. The fact that the slope is slightly shallower suggests that the direct biases vary more than the indirect biases.

**Figure 6:**
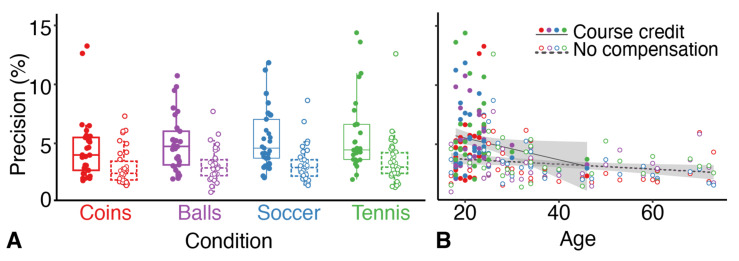
The precision of judgements for the two groups of participants. Each datapoint represents one participant in one condition. Filled symbols indicate students that participated in exchange for course credit; open symbols indicate volunteers who did not receive any compensation. (**A**) Precision for each condition. (**B**) Precision as a function of age for both groups, with for each a linear fit (line) with 95% confidence interval (grey area’s).

In addition to these analyses that we performed to answer our questions, we explored whether there were other interesting patterns visible in the data (Figure 6). Precision generally improved slightly with age for both groups; overall the decrease was 0.092 ± 0.085 and 0.021 ± 0.016 %/year (mean ± 95% confidence interval). The most interesting effect was that an additional analysis showed that the median precision in students that performed the experiment in exchange for course credit was poorer (4.6%) than that of the volunteers who did not receive any compensation (2.9%), even when considering their age.

## 4. Discussion

We compared the judgement of size of images of common objects. We found that the depicted object can influence the perceived size of its image. For balls, we observed an effect that can be interpreted as assimilation towards the familiar size of the depicted objects, whereas for coins we found a contrast effect of familiar size. The participants’ precision was correlated across all conditions, but the bias was uncorrelated: the participants who had larger biases for balls did not necessarily also have larger biases for coins.

This study has a few important limitations. The most serious one is that we only used two sets of familiar objects (balls and coins) with only two objects per set. Consequently, especially since we find completely different results for the two kinds of objects, we cannot be sure that familiarity with the size of such objects is the factor that is causing the observed effects. Other attributes such as color, saturation, brightness, and texture differed as well. And indeed, objects are perceived as larger if they are brighter [31], more saturated [32], and yellow rather than black/white [33,34]. As these effects would all predict that the image of a tennis ball would appear larger than that of a soccer ball, such low-level differences between the images cannot explain the large assimilation to familiar size that we found for the balls. For the rest of the discussion, we will therefore assume that the effect on size is indeed due to familiarity with the size of these objects. However, we cannot exclude the possibility that some other, unidentified low-level factor is responsible for the results.

We originally designed the experiment with the idea that judgments about the size of grey discs would be unbiased. However, the evident correlation that we observed between the biases in the Tennis and Soccer condition (Figure 3B) suggests that judging the size of the unfamiliar grey discs contained the largest idiosyncratic biases of all images used. Indeed, we have shown earlier in judgements of distance that participant assume a certain real-world size for unfamiliar objects [35,36]. Consequently, we cannot exclude the possibility that a prior is involved in judging the size of the unfamiliar grey disc. Such involvement of a prior despite the object being unfamiliar is consistent with finding that precision was not consistently better for the condition with two familiar objects (Balls) than for the conditions with a single familiar object Soccer and Tennis; Figure 4). Moreover, the fact that this prior is not restricted by the known size of the object might explain why the variability in the bias was larger for the condition that involved grey discs (Soccer and Tennis). And lastly, given that we are not sure that the grey disc is perceived without a bias, we cannot draw any conclusion from the positive bias in the Tennis condition. This latter limitation is further enhanced by the limitation that we don’t know what fraction of the participants used a screen size of less than 19” (which we assumed for determining the sign of the effect).

We expected that the effect of familiar size would be larger for the balls than for the coins because the difference in size between the objects themselves is larger. This is indeed the case. However, we did not predict that the effect would switch sign. Why might it do so? There are many aspects in which these two conditions differ. An obvious difference is the shape: sphere or disk. Especially when viewed at close range, a sphere has a larger image size than a flat disk with the same diameter and its center at the same distance, because the outline of the image of a sphere is determined by the tangent to the viewing angle at a closer position than the sphere center (i.e.: you see less than half of the ball). However, as this larger size is based on elements that are at a closer distance, it is unclear how this will affect judging the size of an image. Another possibility is that despite clearly being displayed on the same screen, one assigns the image plane independently to the front of each depicted object, thus considering a larger sphere to be further away. That would make the soccer ball look larger, as is found. Of course, this requires recognizing that a soccer ball is larger than a tennis ball, so it does not change the fact that familiar size is relevant. It could explain the absence of an effect for the coins, but not the observed opposite effect. Yet another option is that the actual coins are smaller than the images for most standard screen sizes, whereas real tennis and especially soccer balls are larger than the images we used. This difference might be especially relevant for this special issue of *Vision*. Coins are generally picked up using a precision grip, tennis balls using a one-handed power grip, and a soccer ball using two hands [37]. When viewing the images, one might automatically associate the depicted items with the corresponding action, which in turn might influence one’s percept [38,39]. The association between grip and perceived size might contribute to the difference between the balls (i.e., the negative bias for Soccer, but positive for Tennis), but not between the coins.

This study examines how various estimates are combined. The Bayesian and anti-Bayesian predictions are independent of the mismatch between image size and known size. In the design of the experiment, we therefore did not consider how a potential mismatch between familiar size and depicted size would influence behavior, as it has been shown to do for reaction times (in a way resembling the Stroop effect [40,41,42]). To maximize precision, we gave our participants all the time they needed to generate a response. Therefore, we cannot directly relate our experimental results to the Stroop-like interference-effects of familiar size.

In an exploratory analysis (Figure 6) we found that students who performed the experiment in exchange for course credit performed worse than participants that were recruited from the network of the second author. This difference might be related to the motivation to produce good results. Although the students performed worse, their performance was not extremely bad. It was close to the level that was reported by the studies mentioned in the introduction [27,29]. Nevertheless, this finding suggests that one should consider the motivation when evaluating precision.

We observed biases in the perception of the size of images of well-known objects. These biases were consistent across participants but differed between images of a soccer ball and smaller objects. We also observed biases in the perception of the size of a grey disk, but this bias differed considerably across participants. These results can be explained by assuming that the size of images of natural objects is biased due to a common prior expectation, whereas the expected size of a grey disk is idiosyncratic. However, it is still unclear why familiar size influences judged size in different directions for different kinds of objects.

## Data Availability

The raw data of this study is available at https://osf.io/8fw3b/ (accessed on 22 February 2022).
